# Versatility of the pedicled buccal fat pad flap for the management of oroantral fistula: a retrospective study of 25 cases

**DOI:** 10.1186/s40902-019-0229-x

**Published:** 2019-11-25

**Authors:** Jinyoung Park, Byung-do Chun, Uk-Kyu Kim, Na-Rae Choi, Hong-Seok Choi, Dae-Seok Hwang

**Affiliations:** 1 Department of Oral and Maxillofacial Surgery, School of Dentistry, Pusan National University, Yangsan, Republic of Korea; 2 Dental and Life Sciences Institute, Pusan National University, Yangsan, Republic of Korea; 3 Dental Research Institute, Pusan National Dental Hospital, Yangsan, Republic of Korea

**Keywords:** Oroantral communication, Oroantral fistula, Buccal fat pad graft

## Abstract

**Purpose:**

Maxillary bone grafts and implantations have increased over recent years despite a lack of maxillary bone quality and quantity. The number of patients referred for oroantral fistula (OAF) due to implant or bone graft failure has increased, and in patients with an oroantral fistula, the pedicled buccal fat pad is viewed as a robust, reliable option. This study was conducted to document the usefulness of buccal fat pad grafts for oroantral fistula closure.

**Materials and methods:**

We retrospectively studied 25 patients with OAF treated with a buccal fat pad graft from 2015 to 2018. Sex, age, OAF location, cause, duration, presence of systemic disease, smoking, previous dental surgery, and side effects were investigated.

**Results:**

A total of 25 patients were studied. Mean patient age was 54.8 years, and the male to female ratio was 19:6. Causes of oroantral fistula were cyst enucleation, tumor resection, implant removal, bone graft failure, and extraction. Excellent results were obtained in 23 (92%) of the 25 patients. In the other two patients that both smoked, a small fistula was observed during follow-up. No recurrence of oroantral fistula was observed after 2 months to 1 year of follow-up.

**Conclusions:**

The incidence of oroantral fistula is increasing due to implant and bone graft failures. Oroantral fistula closure using a pedicled buccal fat pad was found to have a high success rate.

## Introduction

Oroantral fistula (OAF) is mainly caused by extraction or illness, and the treatments used to address it depend on fistula size [1, 2]. Small OAFs (< 3 mm) heal naturally over 1–2 weeks, but surgical intervention is needed for OAFs larger than 3 mm. Surgical intervention may include an advanced buccal flap and a rotational palatal flap, but a pedicled buccal fat pad (BFP) graft or skin grafts may be required for OAFs larger than 5 mm, which are often associated with an inflammatory condition [3]. Numbers of OAF patients are increasing in-line with increases in maxillary bone graft and implant placement procedures [4]. OAF closure is often attempted in local dental clinics, but not uncommon failures result in referrals. Local flaps such as an advanced buccal flap and a rotational palatal flap can be used to treat OAFs of < 5 mm, but a BFP graft is indicated when a larger flap is required for OAFs larger than 5 mm [5].

BFP graft is an established method that has been widely used since 1976 when it was first described by Egyedi [6]. Anatomically, BFP consists of four extensions of the central body, that is, buccal, pterygoid, pterygopalatine, and temporal extensions. According to reports [7, 8], closure of a defect of up to 60 × 50 × 30 mm is possible with a 6-mm-thick BFP of mean volume 10.2 ml for males and 8.9 ml for females and mean weight 9.7 g. BFP grafts fully epithelialize 6 weeks after placement, and the procedure used is straightforward and has a high success rate [9].

The purpose of this study was to document the usefulness of BFP and to identify its indications, side effects, and disadvantages by retrospectively studying the medical records of OAF patients. Furthermore, we determined the proportion of OAFs with an iatrogenic etiology and suggest means of avoiding such problems.

## Materials and methods

The medical records of 25 OAF patients treated by BFP at the oral surgery department of the Dental Hospital of Pusan National University between 2015 and 2018 were reviewed retrospectively. Treatment outcomes were evaluated based on follow-up findings (Table 1). Sex, age, symptom, OAF location, cause, duration, presence of systemic disease, smoking, previous dental surgery, and side effects were investigated. Fisher exact test was undertaken in order to identify associations between different variables and post-operative complication. A *P* value < 0.05 was considered significant. This study was approved by the institutional review board of the hospital and adhered to the Declaration of Helsinki (PNUDH-2018-042).

**Table 1:** Summary of clinical details

Patient	Gender	Age	Chief complain	Site of the defect	Past medical history	Smoking	Length of time OAF present		Etiology	Past dental history
1	F	60	Cystic lesion on Lt. Mx.	#28	Rhinitis	n	4 months	Post-op.	Cyst enucleation	Odontogenic keratocyst on Lt. Mx.
2	M	56	Bone graft	#16,17, #25,26,27	Hypertension	n	3 months(Rt.) 3 months(Lt.)		Bone graft (Rt.) Implant removal (Lt.)	1st operation: OAF closure/c buccal advanced flap on Lt. Mx. &/c buccal fat pad flap on Rt. Mx. 2nd operation: Lt. recurrence OAF closure /c buccal fat pad flap on Lt. Mx.
3	M	37	Cystic lesion on Lt. Mx.	#28	n/s	n	n	Concurrent OAF closure	Excision	Ameloblastoma on Lt. Mx.
4	M	64	Liquid leak through nose Sensation of air rushing	#16	ESS (10 years ago)	n	3 months		Extraction	
5	M	56	n/s	#16-18	Prostate cancer (bone metastasis–Zometa inj. Hx.) DM	n	1 year 10 months	Post-op.	Osteomyelitis	Curettage on Rt. Mx.
6	M	22	Pain, pus discharge, sensation of air rushing	#28	Depression	n	2 years	Concurrent OAF closure	Cyst enucleation CL’s op	Odontogenic keratocyst on Lt. Mx.
7	M	55	Discomfort	#28	n/s	34 PY	n/s	Concurrent OAF closure	Cyst enucleation	Cyst enucleation + oaf closure Dentigerous cyst of #28
8	M	41	Sensation of air rushing	#18	n/s	n	1 month 10 days	Post-op.	Extraction	
9	M	58	Swelling	#28	BPH Insomnia Rhinitis	25 PY	10 days	Post-op.	Cyst enucleation	Mucous retention cyst
10	F	76	Foul odor	#26	Osteoporosis DM	n	3 months		Osteomyelitis	
11	F	59	Discomfort	27	Hypertension Hyperlipidemia Insomnia	n	n/s	Concurrent OAF closure	Excision	Complex odontoma
12	M	49	Pus discharge Sensation of air rushing	#17	n/s	20PY	4 years 6 months		Extraction	
13	M	46	Pus discharge	#16	Hypertension	Stop smoking	1 year		Extraction	
14	M	54	Mobility of #16 Pus discharge	#16	C-L’s op. (20 years ago)	15 PY	1 years	Concurrent OAF closure	Cyst enucleation	Postoperative maxillary cyst
15	M	59	Pain	Rt. Mx.	ESS (20 years ago)	n	2 months	Post-op.	Cyst enucleation	
16	M	46	Pain Liquid leak through nose Sensation of air rushing	Rt. Mx.	n/s	26 PY	6 months		Bone graft Implant removal	Lt. buccal adv. flap failure
17	M	57	n/s	#28	Hypertension DM	n/s	n/s	Concurrent OAF closure	Cyst enucleation	
18	M	78	Bleeding Swelling Pus discharge	Both Mx.	Hypertension Osteoporosis	n	6 months	Concurrent OAF closure	Osteomyelitis	
19	M	70	Nasal congestion Epistaxis Headache	Rt. Mx.	BPH Fatty liver Cerebral aneurysm	n/s	3 months		Implant removal	
20	F	48	Pus discharge Fistula	#16,17	HBV carrier	n	1 months		Implant removal	Buccal adv. flap failure 3times
21	M	61	Liquid leak through nose	#15 buccal gingiva	CL-op (30 years ago, 7 months ago)	20 PY	7 months		C-L’s op	
22	M	65	Foul odor	Lt. Mx.	Hypertension DM Gastritis Hepatitis B	30 PY	1 month		Implant removal	Rotational flap failure
23	F	28	Liquid leak through nose	#17	n/s	n	1 month		Extraction	
24	M	61	Headache Nasal congestion Pus discharge Pain	#14, 16 ,26	DM Hypertension	n	7 months		Implant removal	
25	F	64	n/s	#27	Hypertension fatty liver Osteoporosis	n	1 months		Extraction	

*Mx* maxilla, *DM* diabetes mellitus, *ESS* endoscopic sinus surgery, *inj* injection, *hx* history, *BPH* benign prostatic hyperplasia, *PY* pack years, *adv* advancement, *HBV* hepatitis B virus

**Fig. 1:**
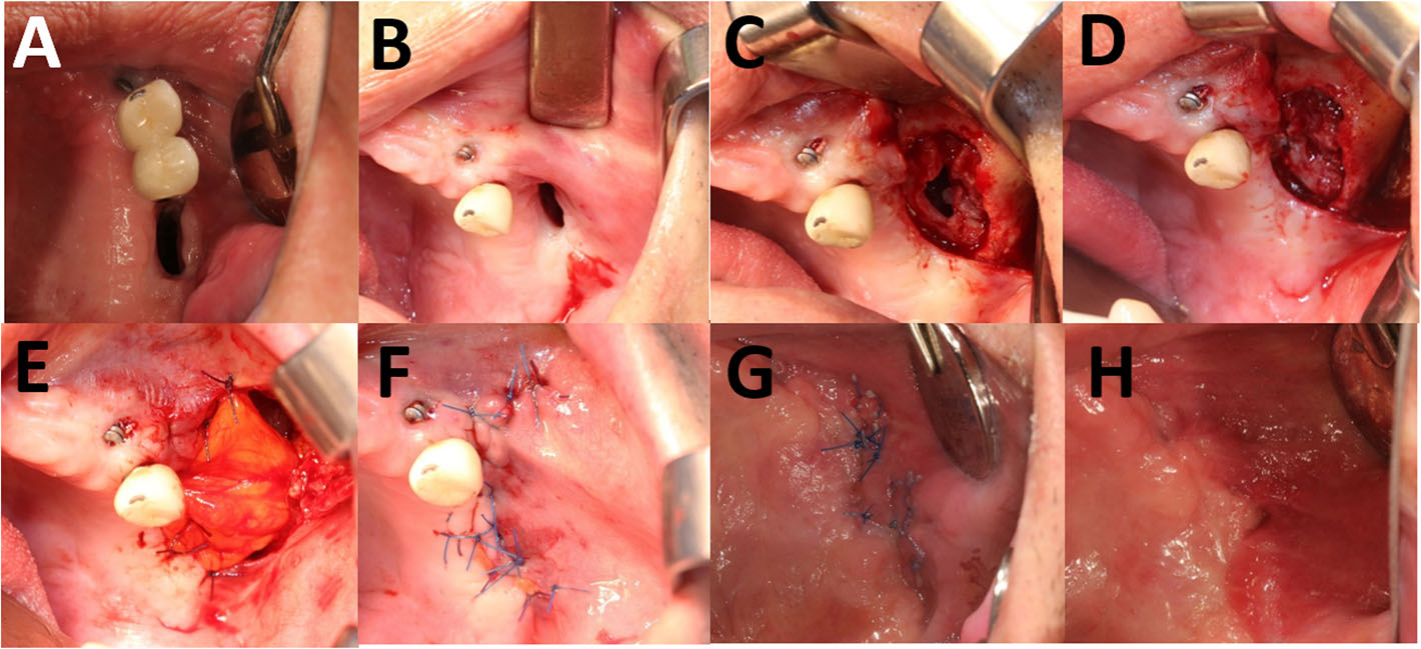
The two-layer technique using a BFP and a buccal advancement flap. **a** At the first visit. **b** Pre-operative state. **c** After reflection of buccal gingiva. **d** After suture of sinus membrane. **e** Buccal fat pad graft on bony defect with suture. **f** After advanced buccal flap suture. **g** 1 week post-operative state. **h** 2 week post-operative state

**Fig. 2:**
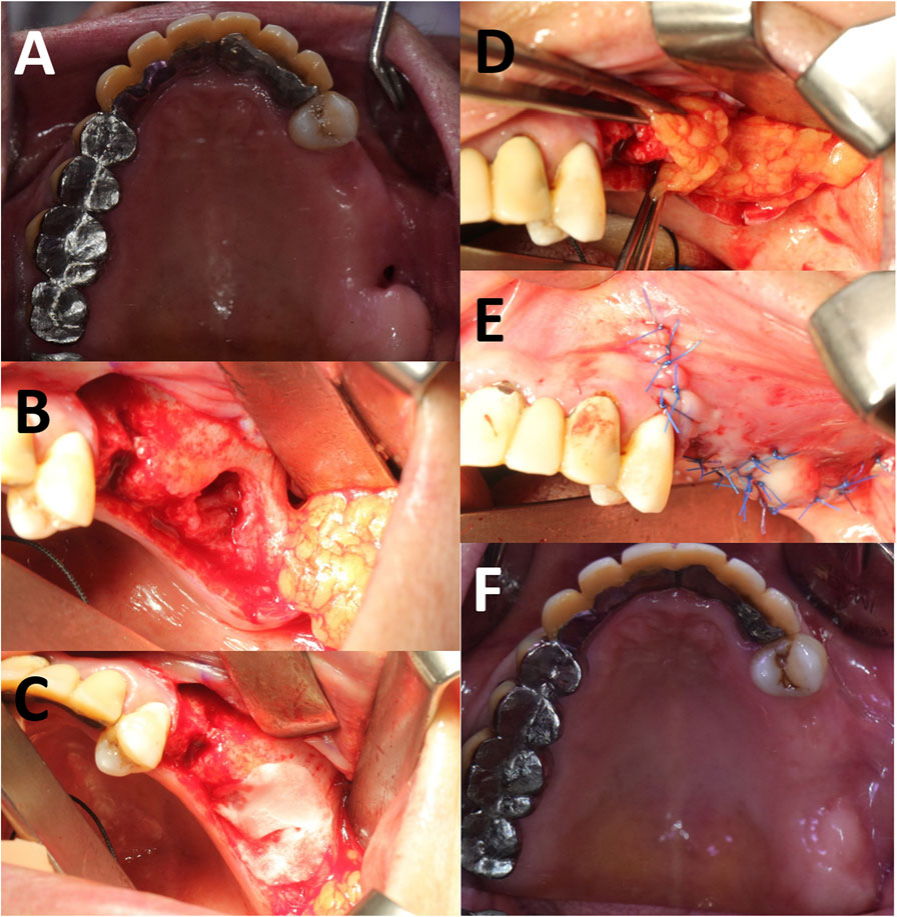
The three-layer technique using collar tape, a BFP, and a buccal advancement flap. **a** Pre-operative state. **b** Bony defect. **c** Collagen paper was applied on the bony defect. **d** Pedicled buccal fat pad was retracted. **e** Post-operative state, **f** 2 weeks after operation

Surgery was performed by a single oral maxillofacial surgeon under general anesthesia or local anesthesia. Twenty-two cases were closed in two layers using a BFP and a buccal advancement flap (Fig. 1). In three cases, collar tape and the two-layer technique was used (Fig. 2). Patients were followed for at least 2 months. All received antibiotics for a month after surgery and were instructed on postoperative care and potential problems.

## Results

Twenty-five patients of mean age 54.8 ± 13.2 years (male to female ratio 19:6) were studied. The prevalence of OAF was greatest in the fourth decade of life (32%). No patient had a specific underlying disease. OAF causes were benign tumor resection or cyst enucleation (9 cases), implant or bone graft failure (6 cases), extraction (6 cases), osteomyelitis (3 cases), and Caldwell-luc (C-L) operation (1 case). Among nine cases of benign tumor resection or cyst enucleation, six cases were prophylactic reasons. In prophylactic cases, the OAF closure operation was successful. Without those prophylactic cases, main reasons of oaf were implant or bone graft failure (31.5%) and extraction (31.5%). Three patients (12%) had a history of failed OAF closure surgery with a buccal advanced flap.

Treatment was satisfactory for all patients and BFP grafts epithelized without side effects. Best results were obtained in 23 (92%) of the 25 patients. In the remaining two cases, a small fistula occurred, but patients did not have discomfort. Both of these patients were smokers and fistulas were detected at 6 and 12 months postoperatively. In one case, healing was achieved after primary closure. In the other, small fistula was healed by itself without any surgical treatment such as primary closure. No necrosis or local inflammation was observed in any patient. Fisher exact test was undertaken in order to identify associations between different variables and post-operative complication. The results of the Fisher exact test did not show a statistically significant association with variables (Table 2).

**Table 2:** Association between risk factors and postoperative complication

		Yes	No	*P* value
Etiology	Implant BG failure	0	17	1.000
	Others	2	6	
Gender	Male	2	17	1.000
	Female	0	6	
Smoking	Yes	2	5	0.070
	No	0	18	

In all patients, BFP epithelialization was complete at ~ 6 week postoperatively. No side effect such as hollow cheek or opening limitation occurred.

## Discussion

A pedicled buccal fat pad flap graft was found to provide a high success rate of oroantral fistula closure in the present study, which concurs with the findings of several other studies [1, 5, 7–10]. The high success rates of BFP flaps are attributed to a rich blood supply [11, 12] from the maxillary artery (buccal and deep temporal branches), superficial temporal artery (transverse facial branch), and facial artery (small branches).

In a previous study, the main cause of OAF was tooth extraction [10], whereas in the present study, the main cause was cyst enucleation or benign tumor resection; we ascribe the difference to the fact that the present study was conducted at a university hospital. The second-most common cause was tooth extraction and the third-most was implant or bone graft failure. Interestingly, unlike previous reports, implantation and extraction contributed equally to OAF in our cohort. Implantation and bone grafting are now being widely applied, and thus, the number of patients with maxillary discomfort due to maxillary implant or bone graft failure [4, 13] and the number of oroantral fistula cases caused by implants and bone graft failures continue to increase.

Interestingly, two patients with bilateral OAF attributed to implants or bone graft failures were treated by BFP on right sides and a buccal advanced flap on left sides, because of smaller OAF sizes on left sides. Unfortunately, after a few weeks, both patients experienced left side OAF recurrence. Closure was achieved by BFP in both, and subsequently, OAF did not recur in either patient. In addition, three patients with OAF caused by implant failure experienced buccal advanced flap failure and were successfully treated by BFP. Based on these experiences, we are inclined to recommend BFP as the treatment of choice for OAF caused by implant failure, but further research is required.

The influences of the effects of age or sex on BFP volume have not been previously studied; accordingly, we advise that before a pedicled BFP flap is used for OAF closure, individual BFP volume be calculated from radiographic images (e.g., CT or MR) to assess whether coverage is possible. Also, additional studies are needed to determine the maximum volume that can be harvested based on considerations of gender, age, and individual variations.

The major limitation of the present study is that it was conducted using a retrospective design. Although all variables in medical records were carefully examined, the possibilities of inaccurate and misleading records cannot be ruled out. Furthermore, our results reveal associations and not causal relations between variables. Given that the numbers of implant and bone graft associated procedures are likely to increase further, we suggest an approach other than a buccal advanced flap and a palatal rotational flap be used to treat OAF. Despite the high success rate of BPF grafting, randomized controlled trials are needed on the topic as the amount of research performed to date is limited.

## Conclusion

The present study confirms that BFP provides a comfortable and reliable means of treating OAF, which is now being treated in large numbers as a result of maxillary implant and bone graft failures. Our experiences lead us to recommend a pedicled buccal fat pad graft to treat for OAFs caused by implant failure and bone graft failure because of its high success rate.

## Data Availability

Not applicable.

## Abbreviations

BFP: Buccal fat pad
C-L: Caldwell-luc
CT: Computerized tomography
MR: Magnetic resonance
OAF: Oroantral fistula
